# Germline *BAP1* Inactivation Is Preferentially Associated with Metastatic Ocular Melanoma and Cutaneous-Ocular Melanoma Families

**DOI:** 10.1371/journal.pone.0035295

**Published:** 2012-04-24

**Authors:** Ching-Ni Jenny Njauw, Ivana Kim, Adriano Piris, Michele Gabree, Michael Taylor, Anne Marie Lane, Margaret M. DeAngelis, Evangelos Gragoudas, Lyn M. Duncan, Hensin Tsao

**Affiliations:** 1 Wellman Center for Photomedicine, Massachusetts General Hospital, Boston, Massachusetts, United States of America; 2 Retina Service, Massachusetts Eye and Ear Infirmary, Boston, Massachusetts, United States of America; 3 Division of Dermatopathology, Department of Pathology, Massachusetts General Hospital, Boston, Massachusetts, United States of America; 4 Massachusetts General Hospital Cancer Center, Massachusetts General Hospital, Boston, Massachusetts, United States of America; 5 Department of Ophthalmology and Visual Sciences, University of Utah School of Medicine, John A Moran Eye Center, Salt Lake City, Utah, United States of America; 6 Department of Dermatology, Massachusetts General Hospital, Boston, Massachusetts, United States of America; University of Connecticut Health Center, United States of America

## Abstract

**Background:**

*BAP1* has been shown to be a target of both somatic alteration in high-risk ocular melanomas (OM) and germline inactivation in a few individuals from cancer-prone families. These findings suggest that constitutional *BAP1* changes may predispose individuals to metastatic OM and that familial permeation of deleterious alleles could delineate a new cancer syndrome.

**Design:**

To characterize *BAP1*'s contribution to melanoma risk, we sequenced *BAP1* in a set of 100 patients with OM, including 50 metastatic OM cases and 50 matched non-metastatic OM controls, and 200 individuals with cutaneous melanoma (CM) including 7 CM patients from CM-OM families and 193 CM patients from CM-non-OM kindreds.

**Results:**

Germline *BAP1* mutations were detected in 4/50 patients with metastatic OM and 0/50 cases of non-metastatic OM (8% vs. 0%, p = 0.059). Since 2/4 of the *BAP1* carriers reported a family history of CM, we analyzed 200 additional hereditary CM patients and found mutations in 2/7 CM probands from CM-OM families and 1/193 probands from CM-non-OM kindreds (29% vs. 0.52%, p = .003). Germline mutations co-segregated with both CM and OM phenotypes and were associated with the presence of unique nevoid melanomas and highly atypical nevoid melanoma-like melanocytic proliferations (NEMMPs). Interestingly, 7/14 germline variants identified to date reside in C-terminus suggesting that the BRCA1 binding domain is important in cancer predisposition.

**Conclusion:**

Germline *BAP1* mutations are associated with a more aggressive OM phenotype and a recurrent phenotypic complex of cutaneous/ocular melanoma, atypical melanocytic proliferations and other internal neoplasms (ie. COMMON syndrome), which could be a useful clinical marker for constitutive *BAP1* inactivation.

## Introduction

For ocular melanoma (OM), monosomy 3 is one of the most powerful independent predictors of metastasis and negative outcome [1]. Recently, somatic mutations of *BAP1* (BRCA1 associated protein-1/ubiquitin carboxy-terminal hydrolase) were identified in a large fraction of high-risk (ie. class 2 by expression profiling) OM tumors which exhibited monosomy 3 [2]. Subsequently, five families with deleterious germline *BAP1* mutations were also described including several kindreds with both ocular and cutaneous melanoma (CM) [3], [4], [5]. These studies suggest that germline *BAP1* mutations may orchestrate a metastatic OM program and that transmission of deleterious alleles may engender a mixed CM-OM pedigree, which could serve as a clinical marker of constitutive *BAP1* loss. The context of susceptibility includes CM patients who report a family history of OM since CM is 40-fold more common than its ocular counterpart (incidence: 20.4 vs. 0.51, www.seer.cancer.gov [6]). We thus set out to determine if germline *BAP1* mutations are more prevalent among (i) OM patients with metastatic disease compared to non-metastatic controls and (ii) CM-OM kindreds relative to CM-non-OM families.

We sequenced and identified *BAP1* mutations in 4/50 OM cases with metastases vs. 0/50 OM controls without metastases (8% vs. 0%, p = .059) and 2/7 CM patients from mixed CM-OM families vs. 1/193 CM probands from non-OM families (29% vs. 0.52%, p = .003). Subsequent analysis of 31 additional members from the 2 CM-OM families revealed that all 4 affected members with CM were in fact *BAP1* mutation carriers. In aggregate, there were 6 distinct novel germline *BAP1* variants out of 300 melanoma patients analyzed. Interestingly, half (7/14) of the known germline mutations to date localized to the C-terminus of BAP1 (close to BRCA1 binding region) suggesting that this may represent the critical domain for heritable cancer risk.

## Materials and Methods

### Patients

This study was performed in accordance with a protocol approved by the Institutional Review Boards (IRB) at the Massachusetts General Hospital (MGH) and the Massachusetts Eye and Ear Infirmary (MEEI).

#### Ocular melanoma cohort (MEEI)

All blood samples were obtained between 1992 and 2006. From this time period, we identified 50 patients who donated blood to our melanoma registry who developed metastasis from OM. These “cases” had at least one sample, obtained at the time of diagnosis, available for analysis. We also included 50 OM patients who had no evidence of metastasis. These “controls” were matched on age at diagnosis (±5 years), gender, largest tumor diameter (LTD, ±3 mm), and ciliary body involvement by the tumor. Selected controls also had follow-up times at least as long as the cases to which they were matched in order to minimize misclassification.

#### Genetically enriched cutaneous melanoma cohort (MGH)

Between April 2001 and June 2011, all in-situ and invasive cutaneous melanoma patients evaluated at the Massachusetts General Hospital Pigmented Lesion Center (see details [7], [8]) were screened for hereditary risk as defined by (i) one or more 1st degree relatives with CM or OM or (ii) two or more affected relatives with CM or OM on one side of family or (iii) three or more primary melanomas in the absence of a family history. The presence and number of melanomas for probands were confirmed via pathology reports for all but a small number of cases (<10%, data not shown). Per our IRB-approved protocol, we only pursued medical record confirmation of reported family history if relatives provided prior consent to participate in our study. Details of both cohorts are listed in Table S1.

### Mutational Analysis

DNA from peripheral blood leukocytes was extracted with the Qiagen DNEasy Kit (Qiagen; Valencia, CA). *BAP1* was analyzed using the primer sequences outlined in Table S2. For tumor analysis, *BRAF* exon 15 was sequenced using 5′ TCA TAA TGC TTG CTC TGA TAG GA 3′ (forward) and 5′GGC CAA AAA TTT AAT AAT CAG TGG A 3′ (reverse) while *GNAQ* was sequenced as described [9]. The putative amplicons were size confirmed by gel electrophoresis, treated with ExoSAP-IT (Affymetrix/USB Corporation, Cleveland, OH) and submitted to the institutional sequencing core.

## Results

**Figure 1:**
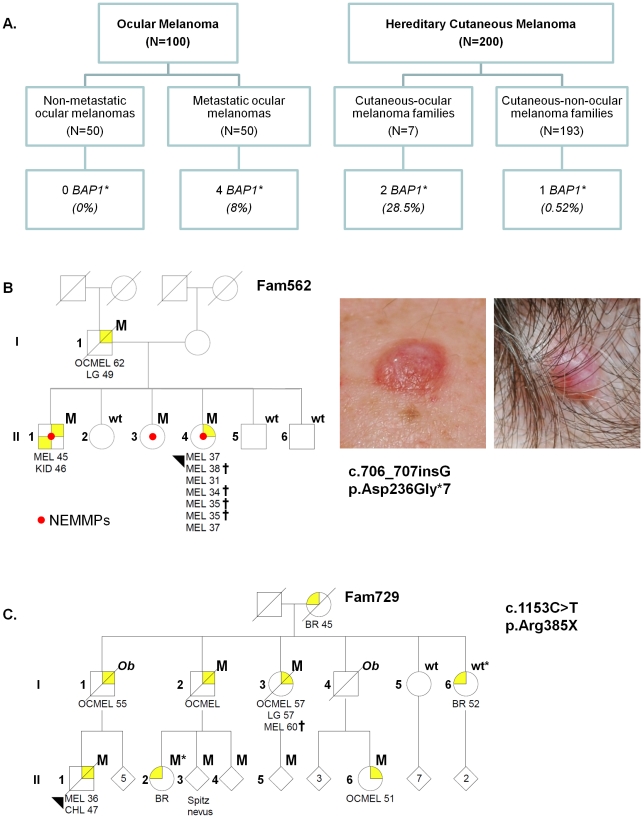
Cohorts and pedigrees. (**A**). Ocular melanoma and cutaneous melanoma cohorts used in this study. (**B**). Fam-562 pedigree and clinical images of two nevoid melanoma-like melanocytic proliferations (NEMMPs) diagnosed in Fam729. (**C**). Fam-729 pedigree. One carrier had a Spitz nevus, which has been reported to harbor somatic *BAP1* mutations [4]. Abbreviations: M, mutation carrier; wt, wildtype germline sequence; Ob, obligate carrier; OCMEL, ocular melanoma; MEL, cutaneous melanoma; NEMMPs, nevoid melanoma-like melanocytic proliferations; BR, breast cancer; CHL, cholangiocarcinoma; LG, lung cancer; KID, kidney cancer; UNP, melanoma of unknown primary site; CNS, central nervous system tumor; LK, leukemia. Crosses indicate CM with a nevoid pattern. The numbers next to the “MEL” indicate ages of diagnosis. *For the sake of confidentiality, the pedigrees have been masked for some non-affected individuals and siblings. Nonessential gender information has also been disguised by a diamond; the number of individuals collapsed into the diamond is indicated.*

Since *BAP1* mutations are known to be more prevalent in high-risk, class 2 OMs, we set out to determine if germline *BAP1* mutations could be instructive of a metastatic phenotype by comparing the rates of *BAP1* alterations among 50 metastatic OM cases and 50 non-metastatic OM controls matched for gender, age, largest tumor diameter (LTD) and ciliary body involvement (Figure 1A; Table S1). The median follow-up duration was 9.2 years for the non-metastatic controls vs. 4.2 yrs for metastatic the cases; thus, misclassification was minimized.

Germline *BAP1* mutations were detected in 4/50 of the metastatic cases (Table 1; #3123: p.Glu611Argfs*5, #2734: p.Ala634Glyfs*5, #3101: p.Lys659X and #3382: p.Arg385X) and in 0/50 of the non-metastatic controls (8% vs. 0%, p = .059, Fisher Exact test). All 4 *BAP1* mutation carriers developed liver metastases and died of their disease. Interestingly, 2 of the 4 metastatic OM patients reported a family history of cutaneous melanoma though neither carrier had cutaneous melanocytic tumors themselves. These findings suggest that *BAP1* alterations may be associated with a more aggressive OM phenotype. Moreover, it supports our initial supposition that germline transmission leads to both CM and OM predisposition, as found in our patients and as previously reported [3], [4].

**Table 1:** BAP1 Mutations Identified In Study.

Individuals Findings							Findings in Family	
ID	Sex/Age	*BAP1* mutation	Primary melanoma	Other melanocytic tumors	Other non-melanocytic tumors	Outcome	Melanocytic tumors	Non-melanocytic tumors
2734	M/58	c.1899_1900ins5; p.Ala634Glyfs*5	Oc mel (choroidal; LTD = 9mm)	None	None	DOD	Cut mel (father)	Bladder (father)
3101	F/53	c.1975A>G; p.Lys659X	Oc mel (ciliochoroidal; LTD = 21mm)	None	Breast lipoma	DOD	Oc mel (cousin)	Kidney *x2* (mat aunts), Bone *x2* (mat aunt, mat uncle)
3123	M/37	C.1831_1834del4; p.Glu611Argfs*5	Oc mel (choroidal; LTD = 17mm)	None	None	DOD	None	Uterine (pat GM)
3382*	F/59	c.1153C>T; p.Arg385X	Oc mel (ciliochoroidal; LTD = 12mm)	None	Lung, DCIS	Alive	Cut mel, Oc mel, Spitz tumor#	Cholangio-carcinoma, breast, lung
562	F/31	c.706_707insG; p.Asp236Glyfs*7	Cut mel (SSM*x2*; **nevoid** *x5*)	NEMMPs (×*10*)	None	Alive	Cut mel, Oc mel#	Kidney, lung
714	M/45	c.178C>T; p.Arg60X	Cut mel (**nevoid**)	NEMMP	None	Alive	Mel (brother)	Lung (mother)
729*	M/36	c.1153C>T; p.Arg385X	Cut mel (SSM)	None	Cholangio-carcinoma	DOD	Cut mel (**nevoid**), Oc mel, Spitz tumor#	Breast, lung

Oc mel = ocular melanoma, Cut mel = cutaneous melanoma, NEMMP = nevoid melanoma-like melanocytic proliferation, SSM = superficial spreading melanoma; DCIS = breast ductal carcinoma-in-situ; DOD = died of disease; mat = maternal; pat = paternal; GM = grandmother.

* found to be part of the same kindred.

# see Figure 1.

To test the possibility that germline *BAP1* inactivation is preferentially linked to a CM-OM familial phenotype, we undertook the complementary strategy and screened 200 hereditary CM individuals for a family history of OM. From this collection, there were 7 CM-OM (7/200 = 3.5%) and 193 (193/200 = 96.5%) CM-non-OM kindreds (Figure 1A, Table S1); thus, mixed CM-OM pedigrees were expectedly uncommon. Germline *BAP1* mutations were detected in 2/7 probands (#562: p.Asp236Glyfs*7 and #729: p.Arg385X) from CM-OM families and 1/193 probands (#714: p.Arg60X) from CM-non-OM kindreds (29% vs. 0.52%; p = .003, Fisher Exact Test). Record tracking afterwards revealed that both carriers of the p.Arg385X mutation (#3382: OM and #729: CM) were indeed members of a single kindred (Fam-729) but were evaluated at MGH and MEEI separately. Proband #714 (p.Arg60X) had only a single sibling who died from metastatic melanoma at age 16 (primary site unknown) and a mother who died from lung cancer when he was young. None of the CM-OM families had germline *CDKN2A*, *ARF* or *CDK4* mutations (data not shown).

Additional genetic material was available in two of the CM-OM kindreds for segregation analysis (Fam-562, 7 total individuals; Fam-729, 26 total individuals). As shown in Figure 1, *BAP1* mutations were identified in all patients with a history of CM, OM or highly atypical melanocytic tumors (see below). Overall, 4/7 members of Fam-562 were carriers of the p.Asp236Glyfs*7 mutation while 9/26 members of Fam-719 harbored the p.Arg385X alteration.

**Figure 2:**
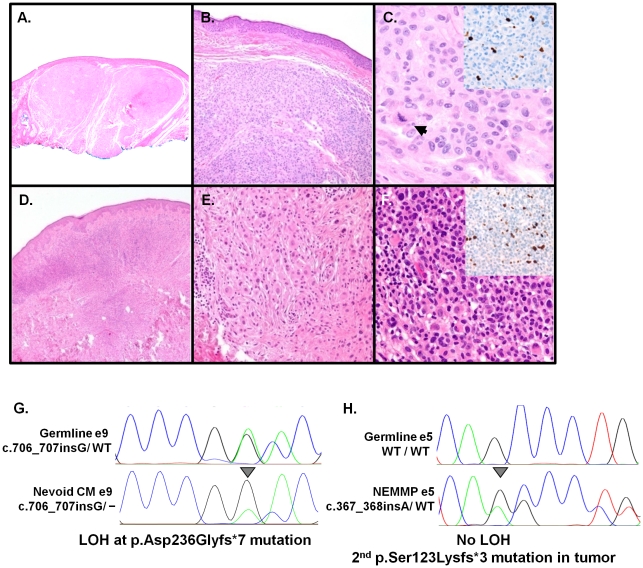
Histologic and molecular analyses of tumors from Fam-562. (**A**)–(**F**): Histology of 2 distinct NEMMPs. (**A**) Scanning view of the first lesion showing two expansile dermal nodules (H&E, 2×,) with a (**B**) benign nevoid appearance (H&E, 10×). (**C**) Atypical cytological features including nuclear pleomorphism and prominent nucleoli and a dermal mitotic figure (arrow) (H&E, 40×) along with focal increases in Ki67 staining (inset). (**D**) In the second lesion, there is an expansile dermal proliferation (H&E, 4×). (**E**) Detail of a field populated by dermal nevic cells with bland nuclear features (H&E,20×). (**F**) A proliferative area showing marked nuclear atypia and hyperchromasia along with elevated Ki 67 staining (inset). Biallelic inactivation of *BAP1* in two tumors through (**G**) loss of the wildtype allele in a nevoid melanoma (ie. LOH; arrow) or (**H**) a secondary mutation (p.Ser123Lysfs*3) in a NEMMP that did not exhibit LOH.

The cutaneous phenotype associated with *BAP1* alterations was quite striking. Carriers from three *BAP1*-mutated families developed “nevoid” melanomas (#714; Fam-562 II-4 and Fam-729 I-1, Figure 1)- an uncommon subtype of CM. We had the opportunity to follow one kindred (Fam-562) over time and examined their skin tumors in detail. In this family, the p.Asp236Glyfs*7 carriers developed diagnostic tumors that were distinctively semi-translucent, orange-red oval-shaped plaques or dome-shaped papules (Figure 1B) rather than the irregularly pigmented plaques and nodules more commonly associated with dysplastic nevi and cutaneous melanomas. However, the banal clinical morphology stood in sharp contrast to the severely atypical histopathology, which harbored cytological attributes reminiscent of a nevoid melanoma. A prominent feature seen in these lesions was the presence of dermal expansile nodule or nodules composed of atypical melanocytes and overall features of nevic cells but with irregular and hyperchromatic nuclei. They also showed rare mitotic activity and focal increases in Ki67 staining (Figures 2A-F). Despite the high degree of atypia, classic features of melanoma such as invasive epithelioid malignant-appearing cells at the base of the tumor and pagetoid spread were not observed. In many cases, the atypical cytological and architectural features fell short of frank malignancy though these lesions clearly lie within the spectrum of nevoid melanomas. We designate these atypical tumors as NEvoid Melanoma-like Melanocytic Proliferations (NEMMPs), which may be related to the Spitz-like tumors reported earlier [4]. Similar histologic features were also evident in non-melanoma tumors removed from p.Arg60X and p.Arg385X mutation carriers. Thus, the spectrum of tumorigenesis from highly atypical NEMMPs to nevoid melanomas appears to be a unique property of *BAP1*-mediated cutaneous carcinogenesis.

Molecular analysis of 7 available cutaneous tumor specimens from Fam-562 (1 superficial spreading melanoma, 1 nevoid melanoma and 5 NEMMPs) revealed loss-of-heterozygosity (Figure 2G) in the nevoid melanoma and a second inactivating somatic mutation (p.Ser123Lysfs*3; Figure 2H) in one of the NEMMPs. In addition, 4 of 7 lesions harbored somatic *BRAF^V600E^* mutations while none had *GNAQ^R183/Q209^* alterations (data not shown). The molecular data support a two-hit event at this locus and suggest that the nevoid melanomas and NEMMPs are more oncogenically aligned with commonly-acquired melanomas and moles rather than blue nevi.

As reported in the literature, the *BAP1* families appear to be at risk for other internal malignancies (Table 1). Among documented carriers, there were two confirmed lung adenocarcinomas (p.Asp236Glyfs*7 and p.Arg385X), one renal cell carcinoma (p.Asp236Glyfs*7), one DCIS (p.Arg385X) and one cholangiocarcinoma (p.Arg385X carrier); unfortunately, genetic material from these other tumors was not available for further analysis. *BAP1* carriers also reported additional kidney, bladder, uterine, breast and bone cancers (Table 1) though these could not be confirmed by pathology since the probands were deceased. There were neither cases of mesotheliomas nor meningiomas.

## Discussion

In this study, we performed a comprehensive screen of *BAP1* in melanoma patients to date and our findings point to an important role for *BAP1* in melanoma predisposition. First, *BAP1* mutations occur with greater frequency among metastatic OM cases compared to non-metastatic OM controls (8% vs. 0%, p = .059). Therefore, germline *BAP1* inactivation appears to contribute to metastatic risk in a small, but significant, proportion of ocular melanoma cases. Biologically, this is consistent with the increased rate of somatic *BAP1* mutations in high-risk OM tumors over low-risk ones (84% vs. 4% [2]).

The higher rate of *BAP1* alterations among CM patients from mixed CM-OM lineages compared to other hereditary melanoma patients (29% vs. 0.52%; p = .003). Observations from our kindreds, along with those already published [3], [4], [5], anchor *BAP1* to a phenotypic complex defined by cutaneous and ocular melanomas, characteristic melanocytic proliferations and other internal neoplasms and thus we propose the designation “COMMON” syndrome, or complex, as a unifying entity. Beyond familial susceptibility, the observed cutaneous histogenesis is rather unique in its formation of an uncommon pattern of nevoid proliferations ranging from frank melanomas to highly atypical dermal expansile nodules (ie. NEMMPs)- a similar entity was described in another recent publication [4]. With case #714, the nevoid CM and NEMMPs were the only clues to the *BAP1* genotype since there were no reports of OM in the family. Interestingly, *BAP1* suppression in uveal melanoma cells led to a profound shift from spindled bipolar cells to more rounded epithelioid cells [2]- a cellular phenotype consistent with the observed histopathology. Taken together, the cutaneous melanocytic tumors are among the most characteristic aspects of the COMMON syndrome and may represent a defining lesion in the future.

**Figure 3:**
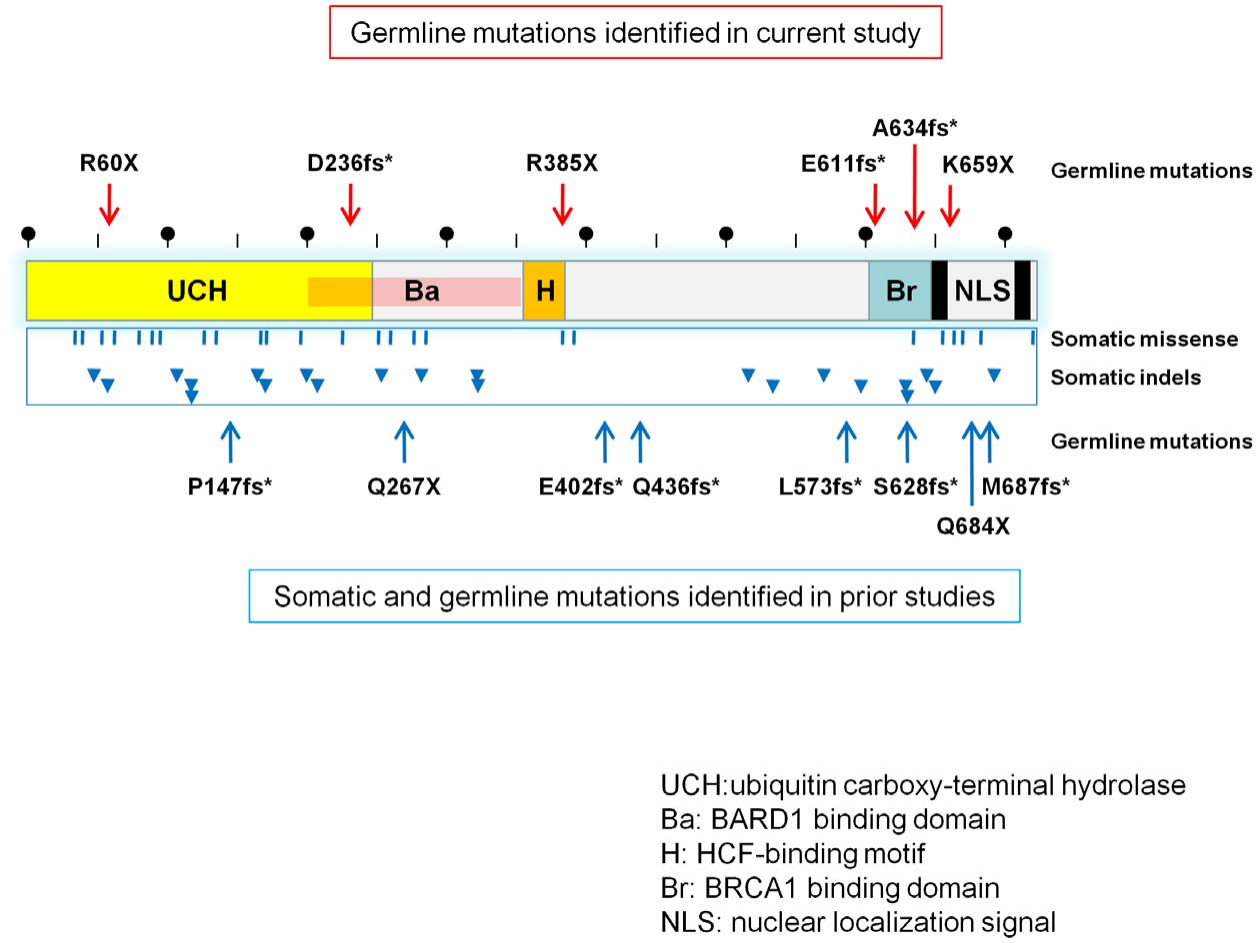
Distribution of *BAP1* mutations. Inactivating germline mutations identified in this study are indicated by the red arrows. Reported somatic missense mutations (blue bars) and indels (blue arrows) from ocular melanoma specimens (COSMIC database; http://www.sanger.ac.uk/genetics/CGP/cosmic/) and germline variants (blue lines) from other families are also shown. Half of the germline variants occur in the terminal 150 amino acids while the somatic changes are more scattered.

When germline mutations from this study are combined with those previously published (Figure 3), there is the suggestion of clustering at the C-terminus of BAP1; this is the region known to interact with BRCA1. This raises the possibility that the BAP1-BRCA1 interface is important for tumor suppression though melanoma is not usually considered a canonical tumor within the *BRCA1* cancer spectrum and *BRCA1* has not been shown to be significantly mutated in either CM or OM [10]. An alternative explanation for the C-terminus clustering is that retention of N-terminus BAP1 function is in fact essential for survival. Since several CM-OM families lack *BAP1* mutations, other components of this pathway may be targeted- a hypothesis that is currently under investigation. Regardless, the low rate of somatic *BAP1* alterations itself in sporadic CM [4] along with the rarity of the characteristic NEMMP and nevoid melanoma lesions in the general melanoma population suggest that *BAP1* mutagenesis is linked to a restricted carcinogenic pathway in melanocytes.

It is also clear that other non-melanocytic tumors may develop within this COMMON context though the full spectrum of susceptible malignancies is still unknown. Among our families, there were three individuals with lung cancer (not mesotheliomas), two of whom carried documented *BAP1* mutations. In recent findings, *BAP1* has also emerged as both somatic and germline targets in mesotheliomas [5], [11] though mesotheliomas were not reported by any of our carriers. Interestingly, patients with OM and mesotheliomas have been described as early as 1972 [12]. Overall, CMs, OMs and mesotheliomas appear to be sentinel cancers within the COMMON spectrum although other cancers are likely to be implicated as more mutations are described.

Functionally, BAP1 is a nuclear deubiquitinating enzyme that has tumor suppressive activities in conjunction with and also independent of BRCA1 [13]. BAP1 has been found to be important in cell cycle regulation and was shown to partner with the transcriptional regulator host cell factor 1 (HCF-1) [14]. With *BAP1* suppression in a uveal melanoma line [2], there were dramatic changes in the levels of genes known to regulate pigment cell development (eg. *EDNRB*, *KIT*, *SOX10*) though the precise mechanism of BAP1 action has yet to be elucidated. Although the directs targets of BAP1 have not been fully elucidated, other melanocyte-specific tumor proteins, such as MITF, have been shown to be regulated by another deubiquitinating enzyme, named ubiquitin-specific protease 13 (USP13) [15]. Furthermore, a deleterious mutation in an yeast deubiquitinating enzyme, Ubp6, has been linked to aneuploidy tolerance [16]. Taken together, the precise targets that drive melanoma progression in the face of BAP1 loss are not known though widespread effects in many diverse pathways are likely given the role of ubiquitination in the maintenance of genetic homeostasis.

In conclusion, our evidence suggests that germline *BAP1* inactivation is preferentially associated with metastatic OM and hereditary CM-OM in a small but significant proportion of cases. The unique clinicopathologic features of the COMMON complex await further clarification with the description of additional families and a more refined genotype-phenotype correlation. Looking ahead, one could envision using COMMON features (eg. nevoid melanomas and NEMMPs) in order to clinically identify potential *BAP1* mutation carriers, who may benefit from targeted screening for high-risk ocular melanomas.

## Supporting Information

Table S1 Characteristics of Study Cohorts. Features of the ocular melanoma and hereditary cutaneous melanoma populations are described.
(DOCX)

Table S2 Primers and conditions.
(DOCX)
