# Supplementary material for: Germline BAP1 Inactivation Is Preferentially Associated with Metastatic Ocular Melanoma and Cutaneous-Ocular Melanoma Families
Source: PLoS One. 2012 Apr 24;7(4):e35295. doi: 10.1371/journal.pone.0035295 (PMC3335872; doi:10.1371/journal.pone.0035295)
Supplement: Table S1 — Characteristics of Study Cohorts. Features of the ocular melanoma and hereditary cutaneous melanoma populations are described. (DOCX) [file pone.0035295.s001.docx]

| **Table S1. Characteristics of Study Cohorts** |  |  |
| --- | --- | --- |
| Ocular Melanoma Cohort (N=100) | | |
|  | OM cases with mets | OM case without mets |
| Gender |  |  |
| Male | 30 | 30 |
| Female | 20 | 20 |
| Mean age | 63.9 (34.9-80.7) | 63.5 (32.4-80.3) |
|  |  |  |
| Ciliary body involved | 22 | 22 |
| Mean largest tumor diameter (LTD) | 14.6 (7.0-21.0) | 14.3 (8.0-22.0) |
| Mean follow-up time | 4.2 (1.0-10.9) | 9.2 (4.4-17.9) |
| Family History Cancer |  |  |
| Yes | 35 (70%) | 35 (70%) |
| No | 14 (28%) | 14 (28%) |
| Unknown | 1 (2%) | 1 (2%) |
|  |  |  |
|  |  |  |
| Hereditary Cutaneous Melanoma Cohort (N=200) | | |
| Gender |  |  |
| Male | 85 |  |
| Female | 115 |  |
| Mean age | 44.3 (12.0-79.0) |  |
|  |  |  |
| Mean number of primary cutaneous melanomas (proband) | 1.58 (1.35) |  |
| Multiple primary melanomas (MPM); no family history | 11 |  |
| Family history of melanoma | 189 |  |
| Additional CM | 179 |  |
| Additional OM | 7 |  |
| Additional extracutaneous melanoma (gastric, vaginal, metastatic) | 3 |  |
| Number of affecteds in family |  |  |
| 1 (MPM) | 11 |  |
| 2 | 118 |  |
| 3 | 44 |  |
| >4 | 27 |  |
