# Supplementary material for: Germline BAP1 Inactivation Is Preferentially Associated with Metastatic Ocular Melanoma and Cutaneous-Ocular Melanoma Families
Source: PLoS One. 2012 Apr 24;7(4):e35295. doi: 10.1371/journal.pone.0035295 (PMC3335872; doi:10.1371/journal.pone.0035295)
Supplement: Table S2 — Primers and conditions. (DOCX) [file pone.0035295.s002.docx]

| **Table S2. Primers and conditions** | |  |  |  |
| --- | --- | --- | --- | --- |
|  |  | Primer | product | Tm |
| Exon1-3 | BAP1-e1F | GTTGTAAAACGACGGCCAGTGTGGGACTGAGGGGC | 495 | 54 |
|  | BAP1-e1R | CACAGGAAACAGCTATGACCGGCCCTGTTCTCTGGG |  |  |
| Exon4 | BAP1-e4F | GTTGTAAAACGACGGCCAGAGTGATGACGCAGTGCAAAG | 303 | 60 |
|  | BAP1-e4R | CACAGGAAACAGCTATGACCCTCCATTTCCACTTCCCAAG |  |  |
| Exon5 | BAP1-e5F | GTTGTAAAACGACGGCCAGTGTCCAGATATGACTGACCTGC | 306 | 60 |
|  | BAP1-e5R | CACAGGAAACAGCTATGACCATGTGGTAGCATTCCCAGTG |  |  |
| Exon6-7 | BAP1-e6F | GTTGTAAAACGACGGCCAGTCTGAAGCTTTGCCTTCCAC | 457 | 60 |
|  | BAP1-e6R | CACAGGAAACAGCTATGACCGCCACTGGGTACCACATACC |  |  |
| Exon8 | BAP1-e8F | GTTGTAAAACGACGGCCAGTGTCTTCCTTCCCACTCCTG | 227 | 60 |
|  | BAP1-e8R | CACAGGAAACAGCTATGACCTGGATACTCTCTGTCCCTCCC |  |  |
| Exon9 | BAP1-e9F | GTTGTAAAACGACGGCCAGCCTGCCAGGATATCTGCCTC | 284 | 60 |
|  | BAP1-e9R | CACAGGAAACAGCTATGACCTCAGAGACAAATGCTGTGGG |  |  |
| Exon10 | BAP1-e10F | GTTGTAAAACGACGGCCAGTTCCTTTAGGTCCTCAGCCC | 318 | 59 |
|  | BAP1-e10R | CACAGGAAACAGCTATGACCAAAAGACTTTCCCTGTTTAGGC |  |  |
| Exon11 | BAP1-e11F | GTTGTAAAACGACGGCCAGTCTCTGGGAAGTGCTGGTTC | 353 | 60 |
|  | BAP1-e11R | CACAGGAAACAGCTATGACCCATGGGAAAATTGCCTGTTG |  |  |
| Exon12 | BAP1-e12F | GTTGTAAAACGACGGCCAGTCTCTGGCTGTGAGTGTCTAGG | 303 | 60 |
|  | BAP1-e12R | CACAGGAAACAGCTATGACCTATCTGCTGCAGGGCATTC |  |  |
| Exon13 | BAP1-e13F | GTTGTAAAACGACGGCCAGAGCCATTCTGGGTACTGCTG | 638 | 60 |
|  | BAP1-e13R | CACAGGAAACAGCTATGACCGAGTGCAGGACACTTTGTGG |  |  |
| Exon14 | BAP1-e14F | GTTGTAAAACGACGGCCAGGAGGGAGGAGGGAAGTGG | 321 | 55 |
|  | BAP1-e14R | CACAGGAAACAGCTATGACCATCAAGAACTTGGCACCTGG |  |  |
| Exon15-16 | BAP1-e15F | GTTGTAAAACGACGGCCAGCTGCCTATTGCTCGTGGG | 438 | 59 |
|  | BAP1-e15R | CACAGGAAACAGCTATGACCCAAGGTCTGCTCAAGCCTC |  |  |
| Exon17 | BAP1-e17F | GTTGTAAAACGACGGCCAGACAGGGAGGGCCATGAG | 298 | 59 |
|  | BAP1-e17R | CACAGGAAACAGCTATGACCTACTGGGAAAAGGGGAAGTG |  |  |
| exon 9 FFPE-1 | BAP1-e9F | GTTGTAAAACGACGGCCAGCCTGCCAGGATATCTGCCTC | 200 | 60 |
|  | BAP1-e9_FFPE-1R | CACAGGAAACAGCTATGACCCCTGCTGCAGAGCCTCTAGT |  |  |
| exon 9 FFPE-2 | BAP1-e9_FFPE-2F | GTTGTAAAACGACGGCCAGTCAACCTGATGGCAGTGGT | 200 | 60 |
|  | BAP1-e9_FFPE-2R | CACAGGAAACAGCTATGACCgaagggaggaggaatgcag |  |  |
|  |  |  |  |  |
| DNA: 50ng, primer 0.4uM, taq 0.5 unit, reaction vol: 20ul | | |  |  |
| PCR condition |  |  |  |  |
| Hotstart Taq (Qiagen) was used to amplify exons 1-3 and exon 14 | | |  |  |
| 94 degC x 15min |  |  |  |  |
| 94 degC x 30sec |  |  |  |  |
| 54/59 degC x 30sec | 35 cycles |  |  |  |
| 72 degC x 30 sec |  |  |  |  |
| 72 degC x 10min |  |  |  |  |
| Rest of the exons/FFPE used TAKARA ex Taq | | |  |  |
| PCR condition: |  |  |  |  |
| 94 degC x 15min |  |  |  |  |
| 94 degC x 30sec |  |  |  |  |
| 59/60 degC x 30sec | 35 cycles |  |  |  |
| 72 degC x 30 sec |  |  |  |  |
| 72 degC x 10min |  |  |  |  |
|  |  |  |  |  |
| PCR reaction with FFPE template | |  |  |  |
| DNA using 1-10ul DNA from 50ul elution, primer 0.4 uM, taq 0.5 unit, 0.6ug/ul BSA | | |  |  |
